# Novel Hybrid Virtual Screening Protocol Based on Molecular Docking and Structure-Based Pharmacophore for Discovery of Methionyl-tRNA Synthetase Inhibitors as Antibacterial Agents

**DOI:** 10.3390/ijms140714225

**Published:** 2013-07-09

**Authors:** Chi Liu, Gu He, Qinglin Jiang, Bo Han, Cheng Peng

**Affiliations:** 1 State Key Laboratory of Biotherapy, West China Hospital, Sichuan University, Chengdu 610041, Sichuan, China; E-Mail: zarbiya@foxmail.com; 2 Department of Pharmacy, Chengdu Medical College, Chengdu 610083, Sichuan, China; 3 State Key Laboratory Breeding Base of Systematic research, Development and Utilization of Chinese Medicine, Chengdu University of Traditional Chinese Medicine, Chengdu 611137, Sichuan, China; E-Mails: hanbo@cdutcm.edu.cn (B.H.); pengchengchengdu@126.com (C.P.)

**Keywords:** pharmacophore, molecular docking, methionyl-tRNA synthetase, virtual screening

## Abstract

Methione tRNA synthetase (MetRS) is an essential enzyme involved in protein biosynthesis in all living organisms and is a potential antibacterial target. In the current study, the structure-based pharmacophore (SBP)-guided method has been suggested to generate a comprehensive pharmacophore of MetRS based on fourteen crystal structures of MetRS-inhibitor complexes. In this investigation, a hybrid protocol of a virtual screening method, comprised of pharmacophore model-based virtual screening (PBVS), rigid and flexible docking-based virtual screenings (DBVS), is used for retrieving new MetRS inhibitors from commercially available chemical databases. This hybrid virtual screening approach was then applied to screen the Specs (202,408 compounds) database, a structurally diverse chemical database. Fifteen hit compounds were selected from the final hits and shifted to experimental studies. These results may provide important information for further research of novel MetRS inhibitors as antibacterial agents.

## 1. Introduction

Translation is one of the most complex biological processes and involves diverse protein factors and enzymes, as well as messenger and transfer RNAs [1]. Many translational factors and enzymes are considered as housekeeping proteins, because this process is required for the basic operation of cells [2]. Aminoacyl-tRNA synthetases (aaRSs) are a class of enzymes that were validated as antimicrobial targets [3]. The enzymatic reaction of aaRS generally consists of the following steps: The recognition of a specific amino acid and ATP, the formation of an aminoacyl-adenylate, the recognition of a specific tRNA and the transfer of the aminoacyl group to the 3′-end of the tRNA [4]. Although the activities of aminoacyl-tRNA synthetases are essential in all living organisms, the selective inhibition of pathogen synthetases over their human cellular counterparts provides an attractive antibacterial mode of action for discovering novel classes of antibiotics, particularly for the treatment of antibiotic-resistant bacterial strains and *Trypanosoma brucei*. In recent years, particular aaRS inhibitors have emerged, including those that are mimics of the aminoacyl adenosine monophosphate intermediate (aa-AMP). Although most of the aa-AMP mimics demonstrated low nanomolar binding affinities against their corresponding aaRSs, they generally displayed weak antimicrobial activity, because of poor bacterial cell permeability [5–11].

Methionyl-tRNA synthetase (MetRS) is one of the aminoacyl-tRNA synthetases, which belongs to the family of class I aaRSs that acylates methionyl-tRNA with methionine. MetRS has an essential function in the core biological process of translating nucleotide-encoded gene sequences into proteins [12]. Comprehensive attempts to search for compounds that can specifically target bacterial MetRSs resulting in the inhibition of bacterial growth have been recently investigated. Jarvest *et al.* reported the discovery and structure-activity relationships of a series of 2-[(aminopropyl)-amino]-4(1*H*)-quinolinones as a new class of *S. aureus* methionyl-tRNA synthetase (SaMetRS) inhibitors with nanomolar IC_50_ values and potent antibacterial activities [3]. Kim *et al.* performed virtual screening (VS) of a chemical database of commercially available compound collections (ChemDiv Inc.) to find the scaffolds of MetRS inhibitors based on the principal pharmacophores of methionyl adenylate [13]. Tandon *et al.* performed high-throughput screening to identify oxazolone-dipeptides that showed selectivity for SaMetRS *versus* human MetRS (hMetRS) [14]. Lee *et al.* reported pyrazole derivatives inhibitors of methionyl-tRNA synthetase (MetRS) by high-throughput screening, which bear modest micromolar inhibiting properties of the bacterial MetRS enzyme from SaMetRS and *Enterococci faecalis* methionyl-tRNA synthetase (EfMetRS), but with weak selectivity to hMetRS [15]. Finn *et al.* identified conditions for crystallizing SaMetRS with small molecule inhibitors by using a high-throughput, low-volume approach to crystallographic screening [16].

Our research group aimed at searching for novel potent antimicrobial compounds [17], and we attempted to explore accurate and reasonable methodology of hybrid structure-based pharmacophore and virtual screening methods. The combined structure- and ligand-based drug design strategy provided insights into the molecular recognition patterns required for MetRS binding and for developing a structure-based pharmacophore model (MCBP) that can be used for VS to discover novel potential lead compounds [18–23]. The structure-based pharmacophore and VS results helped us predict the biological activities of the series compounds with a change in the chemical substitutions and provided useful references for the design of novel MetRS inhibitors. The top 1000 hits from the pharmacophore model-based virtual screening (PBVS) search were next screened with docking-based virtual screenings (DBVS) by docking into the SaMetRS homology model by using Libdock and retrieving ten poses per ligand. The set of docked compounds were then scored using LigScore and ranked based on consensus score. Complexes with the highest computed score representing 150 different compounds were then docked into the hMetRS homology model by using Ligandfit to ensure that the ligand possess both inhibitory efficiency and selectivity to the pathogen. Compounds with low Ligscore in the hMetRS homology model were filtered and analyzed for druglikeness. Fifteen final hit compounds were selected for acquisition and antibacterial testing. The results might be helpful in understanding the inhibitory mechanism and in future discovery of novel antibacterial compounds.

## 2. Result and Discussion

### 2.1. Generation and Validation of Structure-Based Pharmacophore

**Figure 1:**
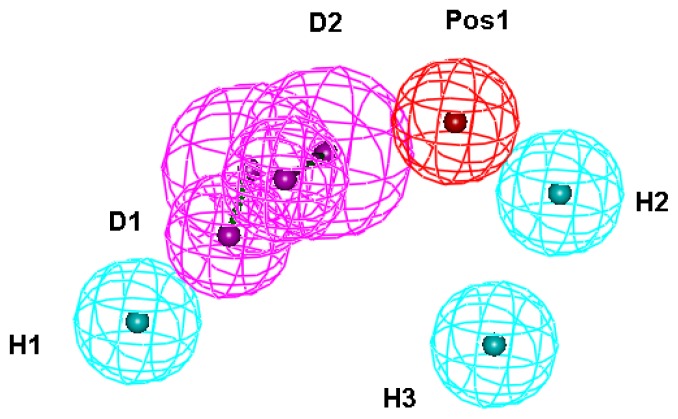
The structure-based comprehensive pharmacophore model for pharmacophore model-based virtual screening (PBVS). Screenshots were taken from Discovery Studio. Features of the pharmacophore models are color-coded as follows: Hydrogen bond acceptor (HBA), green; hydrogen bond donor (HBD), violet; hydrophobic (H), light blue; positive ionizable (pos), red; negative ionizable (neg), blue.

Fourteen X-ray crystallography structures of MetRS in complex with small molecular inhibitors were used to construct the pharmacophore. The results of molecular superposition based on MODELLER [24] are reported below (see Figure S1). The detected pharmacophore features, as well as their statistical frequency, which measures the number of complexes in a given pharmacophore feature, are shown in Table 1 and Figure S2. Nineteen pharmacophore features, including six hydrophobic features (H1–H8), eight hydrogen bond donors (D1–D8), two hydrogen bond acceptors (A1 and A2), two positive ionizable point (Pos1 and Pos2) and one negative ionizable point (neg) exist. Six (H1, H2, H3, D1, D2, and Pos1) out of 18 detected pharmacophore features were found common in the fourteen complexes. We assumed that the pharmacophore features present in the complexes with a high probability were more important than features exhibiting low probability. For a full pharmacophore map, excluded volume features should be included, which reflected potential steric restriction and corresponded to positions that were inaccessible to any potential ligand [18]. Twenty-six excluded volume features were found in the ATP-binding and methionine-binding sites, whose spaces were occupied by residues Pro247, Ile248, Tyr250, Asp287, His289, Gly290, Glu368, Val471, Tyr472, Val473, Trp474, Asp476, Ala477, Leu478, Tyr481, Ile519 and His523. A comprehensive pharmacophore map involving excluded volume spheres is shown in Figure S2. The initial comprehensive pharmacophore map was too restrictive and unsuitable for the virtual screening, because it contained a large number of chemical features, and the fit of a molecule to such a pharmacophore was still out of reach even for current state-of-the-art computational tools. A correctly reduced pharmacophore model is more preferred in terms of practical application [25]. Accordingly, the top ranked six features (H1, H2, H3, D1, D2 and Pos1), which were found to be present in the 14 complexes with more than 60% probability, would be more appropriate in actual practice. These features were consequently selected from the comprehensive pharmacophore map and were merged to generate a structure-based pharmacophore (Figure 1).

**Figure 2:**
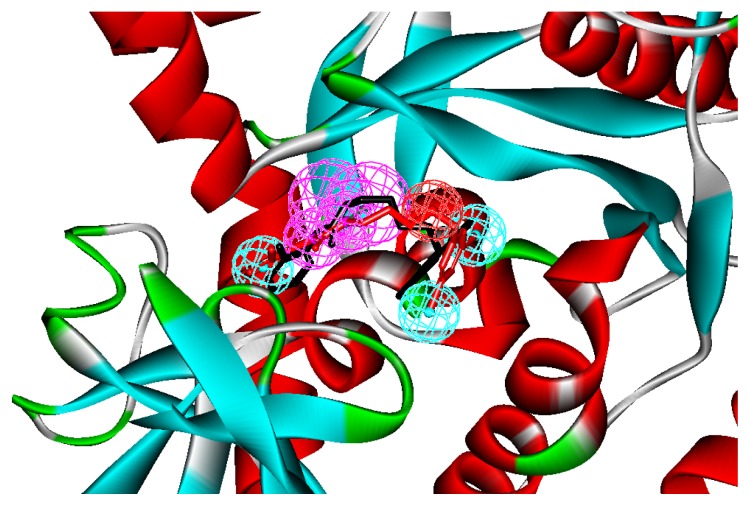
The mapping of the structure-based pharmacophore and the best mapping conformation (red bars) and the bound conformation (black bars) for the ligand to methione tRNA synthetase (MetRS) are superimposed on the pharmacophore model. Screenshots were taken from Discovery Studio. Features of the pharmacophore models are color-coded as follows: Hydrogen bond acceptor (HBA), green; hydrogen bond donor (HBD), violet; hydrophobic (H), light blue; positive ionizable (pos), red.

We applied the method to inhibitor “392,” the co-crystal inhibitor of *T. brucei* MetRS, whose bioactive conformation is known from the co-crystal structure of MetRS and with a binding mode similar to other derivatives to verify if the pharmacophore model can find the correct bioactive conformation. A reliable pharmacophore model may be used to determine the bioactive conformations of the ligands that share the same binding mode. The conformation selected for each compound, assumed as the bioactive conformation, corresponds to the conformation that best fit the pharmacophore. Thus, the X-ray crystal structure of MstRS protein (PDB code: 3U1F) was selected from the Protein Data Bank. The bound conformation of this inhibitor was mapped onto the pharmacophore model by using the flexible fitting method and the best mapping only option in the “Ligand Pharmacophore Mapping” protocol and simultaneously superimposed to the best mapping conformations (Figure 2). The root mean square deviation (RMSD) value between the heavy atom positions of the bound and the best mapping conformation was 0.34 Å. The result showed that the pharmacophore model is capable of reproducing the bioactive conformation from the Protein Data Bank, which supports our choice for bioactive conformation obtained from the best mapping conformation.

### 2.2. 3D-Model Structure of SaMetRS and hMetRS Were Built by Homology Modeling

Experimental crystallology structures for SaMetRS and hMetRS are yet to be made, although we have effective inhibitors for SaMetRS. Therefore, we developed a 3D-model structure by using the homology modeling method. Multiple sequence alignment by using Discovery Studio revealed the conserved amino acids present in the modeled and the template proteins. Figure S3 shows the sequence alignment between the target protein, SaMetRS, hMetRS and the template protein, *E. Coli* MetRS (PDB ID: 1PFY). BLAST (blastp) showed about 32% and 35% sequence identity between the target and template sequences, respectively. The methionine-binding pocket of *E. coli* is formed by key amino acids, such as L13, Y15, H23, D52, W253, A256, Y260, G294, K295, D296, H301 and W305. Most of the residues are conserved in all MetRS. Similar residues, like I12, A270 and E272 of *S. aureus*, were present in place of L13, G294 and D296 residues of *E. coli*, respectively, whereas the rest of the binding pocket residues were all the same [26]. The final homology modeling calculation by using the MODELLER module in Discovery Studio generated a reliable 3D structure of SaMetRS and hMetRS (Figures S4 and S5), because Profile-3D, a protein structure validation module in Discovery Studio, predicted that 91.3% (SaMetRS) and 88.2% (hMetRS) of the residues of the 3D structure lied in the most favored regions, unlike the template protein, which has 87.9% of the residues in the most favored regions. The RMSDs between the template and the target structures were 0.37 Ǻ for SaMetRS and 0.42 Ǻ for hMetRS.

### 2.3. Molecular Docking

Optimization for the docking parameters and scoring functions was done in advance, because docking parameters and scoring functions have an important influence on the performance of molecular docking-based virtual screening. In this study, the Libdock module, Ligandfit module and ChemScore kinase in Discovery Studio were employed for DBVS. The crystal structure of *T. brucei* MetRS complexed with the small molecular inhibitor “392” (PDB ID: 3U1F) was chosen as the reference receptor structure, because it has a high resolution (2.20 Å) and representative inhibitor structures. We adjusted the docking parameters until the docked pose is as close as possible to the original crystallized structure in the binding site of MetRS. The final optimized docking parameters of Libdock mainly consist of the following: (1) The “number of hotspots” set to 200; (2) the docking tolerance set to 0.20; and (3) the “conformation method” set to “FAST”; other parameters were kept on default settings. The docking parameters of Ligandfit used default settings, unless for special statements. For the selection of scoring functions, we chose a set of known MetRS inhibitors whose IC_50_ values span a range of three orders. These inhibitors were docked into the binding site of MetRS, in which the optimized docking parameters were used. Different scoring functions, including GoldScore, ChemScore and a modified ChemScore—An optimized scoring function for the kinase-related docking (hereafter called Chemscore kinase), were calculated. Chemscore kinase gave the best correlation coefficient. Therefore, Chemscore kinase was used in the subsequent DBVS study.

### 2.4. Database Screening

A hybrid virtual screening was performed on the basis of the homology model structures of MetRS and structure-based pharmacophore to identify novel MetRS inhibitors. First, The Specs database was used for the pharmacophore based virtual screening, and 1000 hits were obtained out of the 202,408 compounds, because a preliminary virtual screening test showed that PBVS is faster than DBVS in terms of the screening speed. Second, 150 compounds out of the previous 1000 hits and 25 known MetRS inhibitors from the literature were selected through Libdock and the SaMetRS homology model by using the docking score of the active compound as the cutoff value. These 150 compounds were subsequently filtered by Ligandfit and the hMetRS homology model according to their docking score values calculated in flexible DBVS. Those compounds with high Ligandfit docking score values were excluded from the hit compounds, because of their potential adverse effect on hMetRS. Finally, 15 compounds were visually chosen from the top hits. These compounds must satisfy the following criteria to qualify for the next assays: (1) Good interactions with the key residues in the binding site of MetRS, such as L13, Y15, H21, D296 and H301; (2) a ChemScore kinase value greater than 20; (3) contain scaffolds different from that of the known MetRS inhibitors; and (4) must be easily purchased.

**Figure 3:**
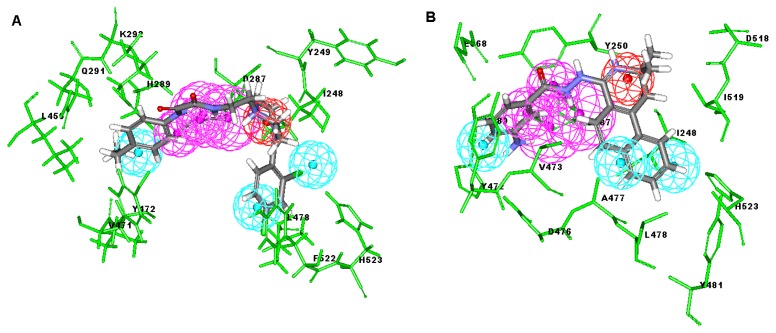
Docked binding models of the two representative compounds. (**A**) ZINC06843697; (**B**) ZINC00729256.

Among these hits, compounds ZINC06843697 and ZINC00729256, which are different in their chemical scaffolds, were identified as promising novel leads against MetRS with good MIC value of pathogenic bacteria and non-cytotoxicity to HEK293 cell. The docked conformations with key residues in the active site of MetRS are shown in Figure 3. The two lead compounds would be focalized for further refining and optimizing to discover novel anti-bacterial agents, because they also satisfied all the drug-like properties.

### 2.5. *In Vitro* Minimum Inhibitory Concentration and Cytotoxicity Assay

The 15 final hit compounds were purchased and screened for preliminary *in vitro* antibacterial activity against our five ATCC-bacterial strain panel (*Staphylococcus aureus* ATCC 29213, Methicillin-resistant *Staphylococcus aureus* ATCC 43300, *E. coli* ATCC 25922, *Pseudomonas aeruginosa* ATCC 27853 and *Klebsiella pneumoniae* ATCC 700603) by using the standard broth dilution method [27,28]. As shown in Table 2, most compounds exhibited antibacterial activities with MIC (minimum inhibitory concentration) ranging from 4 to 64 μg/mL. From the initial results, most compounds were more effective against Gram-negative bacteria, especially *P. aeruginosa*. All of the 15 final hit compounds were tested against HEK293 cell lines to avoid potential cytotoxicity of normal human cells. In the 15 compounds tested, most compounds showed non-cytotoxicity to HEK293 cells, whereas only four compounds inhibited cell growth, with an IC_50_ value ranging from 3 to 20 μM (Table 2). Compounds ZINC02086896 and ZINC04380079 inhibited the proliferation of HEK293 cells with IC_50_ values of 3.15 and 6.84 μM, respectively. Further chemical optimization is being performed to better understand the key residues for activity and selectivity.

## 3. Computational and Experimental Methods

### 3.1. Generation of Structure-Based Pharmacophore Models

A set of fourteen crystal structures of MetRS in complex with diverse ligands (Table 3) were obtained from the Protein Data Bank (PDB) [29–31]. The crystal structures with ATP or methionine, the natural ligand of MetRS, were not used in the analyses to avoid the unnecessary noise that will likely be introduced into the pharmacophore model. Accurately locating the pharmacophore features for their respective cases was difficult and complicated because of the high mobility of water molecules and ions and their less conservative location in the active pocket [20]. Thus, they were all removed from the structures. Any structural issue of the protein and the ligand was carefully examined by visual inspection. The coordinates of all MetRS-inhibitor X-ray crystal structures were transformed into a common reference frame by using the “Multiple Structure Alignment (MODELLER)” module within the Discovery Studio (DS) [32].

**Figure 4:**
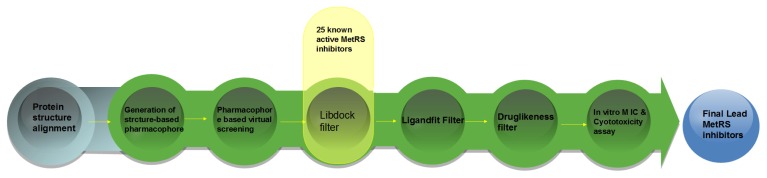
Steps of the generation of the structure-based pharmacophore model and the hybrid virtual screening approach based on structure-based pharmacophore model and molecular docking.

The whole process of generation and utilization of the structure-based pharmacophore models is illustrated in Figure 4 and detailed as follows. The complex-based pharmacophore generation module in Discovery Studio was used to generate fourteen individual complex-based pharmacophore models based on the previously aligned structures. For purposes of creating a structure-based pharmacophore model, all the pharmacophore features identified by Discovery Studio were clustered according to their interaction pattern with the receptor. The cluster centers were identified using the Discovery Studio [32]. The obtained model was further refined by modifying the constraint tolerance of the spheres in accordance with the default values of pharmacophore modules in Discovery Studio.

### 3.2. Pharmacophore-Based Virtual Screening

Virtual screening of chemical databases could aid in finding novel lead compounds suitable for further research. Virtual screening has the advantage over other *de novo* design methods, because hit compounds can be easily obtained from commercial sources for biological activity assay [33]. In the present study, a structure-based pharmacophore model comprising six chemical features was used as a basis for searching Specs chemical database consisting of 202,408 structurally diversified small molecules. We performed all database searching experiments by using the “Best” conformation generation option and the “Flexible” fitting method option in the “pharmacophore” protocols of Discovery Studio software. The molecules in the Specs database that fit on all the features of the pharmacophore model were retained as hits [34,35]. Geometric fit values were calculated for each hit compound based on the quality of mapping chemical substructures on to the pharmacophore features. The criterion for screening for further validation gave high fit values, which indicate good matches.

### 3.3. Homology Modeling

BLAST (blastp) was employed to search relevant targets or template proteins for building SaMetRS and hMetRS protein structures. The “Align Multiple Sequence” module of Discovery Studio was applied to compare the SaMetRS and hMetRS sequences with *E. coli* MetRS protein. The MODELLER module in Discovery Studio was used to build the homology model [24]. Sequence alignments are shown in Figure S3 and the final 3D model was validated by Profile-3D module in Discovery Studio [32].

### 3.4. Molecular Docking Study

All molecular docking studies were carried out by Libdock and Ligandfit modules in Discovery Studio, and the CHARMm force field was used. The homology structures of the SaMetRS and hMetRS were taken as the receptor structures. The binding site was defined as a sphere containing the residues that stay within 12 Å from the co-ligand, which cover the ATP-binding region and methionine-binding region at the active site. The structures of 25 active compounds [3,8–10] from the literature were initially sketched in Discovery Studio and then imported into DBVS. First, following PBVS, the compounds that ranked in the top 1000 in terms of the fit value were selected for DBVS with SaMetRS by using the docking score of the active compound as the cutoff value. Compounds in the top 150 were selected for the following screening. The Ligandfit module of Discovery Studio was then employed to complement the previous DBVS filters with hMetRS homology models. In contrast to Libdock, Ligandfit considered the flexibilities of both ligands and side chains of proteins. Top-ranked compounds of Ligandfit were excluded from the final hits. The DBVS hit compounds were selected and evaluated for their druglikeness properties by using Lipinski’s rule of five ((1) not more than five hydrogen bond donors; (2) not more than ten hydrogen bond acceptors; (3) a molecular weight below 500 Da; and (4) a partition coefficient log P less than five) and ADME/T (absorption, distribution, metabolism, excretion and toxicity) filters in Discovery Studio. The samples of 15 final hit compounds were purchased from the Specs Company and then evaluated via biological assays.

### 3.5. *In Vitro* Minimum Inhibitory Concentration Assay

MICs were determined using a microdilution method with Muellere Hinton Broth (MHB) for *Staphylococci* and Lennox Broth (LB) for *Enterococci*, following the National Committee for Clinical Laboratory Standards (NCCLS) (now called the Clinical Laboratory Standards Institute [CLSI]). The stock solutions of the test compounds were diluted to give a two-fold series, with final chemical concentrations ranging from 64 to 0.0125 μg/mL. The MIC was defined as the lowest concentration of the chemical that inhibited the development of visible bacterial growth after an incubation for 16 h at 37 °C.

### 3.6. *In Vitro* Cytotoxicity Assay

Human Embryonic Kidney 293 cell line (HEK293) was acquired from ATCC. The cells were cultured in DMEM medium (Gibco-BRL, Invitrogen Co. Ltd., Carlsbad, CA, USA) supplemented with 10% (*v*/*v*) fetal bovine serum plus 100 μg/mL amikacin. The cells were maintained in a 37 °C humidified incubator with 5% CO_2_ atmosphere. Analysis of *in vitro* cytotoxicity was performed in the presence of compounds or blank solvent on cell lines seeded in 96-well plates at the density of 2 × 10^3^ cells/well in serum-containing media. After 24 h incubation at 37 °C, the cells were treated with different concentrations of compounds along with a blank solvent. The cells were incubated in these conditions for 48 h. The cell viability was assessed with the 3-(4,5-dimethylthiazol-2-yl)-2,5-diphenyltetrazolium bromide (MTT) method, as previously described [36].

## 4. Conclusions

In this study, a multi-step virtual screening, including structure-based pharmacophore, rigid docking and flexible docking, was used to search for novel MetRS inhibitors from the Specs database. We utilized fourteen crystal structures of MetRS bound to small molecular inhibitors to generate a structure-based pharmacophore. The capability of structure-based pharmacophore model to predict the bioactive conformations and molecular alignments of a wide variety of MetRS inhibitors in the structurally diverse data sets was validated. This work provided an approach to generate a structure-based pharmacophore-guided virtual screening based on a set of crystal structures of protein-ligand complexes. The study suggested that in the search of novel inhibitors, the structure-based pharmacophore-guided virtual screening could be useful in getting the predictive models that may provide useful information required for proper understanding of the important structural and physicochemical features. Furthermore, a hybrid protocol of virtual screening methods, including PBVS, rigid DBVS and flexible DBVS, was introduced in the discovery of MetRS inhibitors. Finally, the hybrid VS approach was applied to screen the Specs chemical databases (202,408 compounds). Fifteen compounds were selected from the final hits and were shifted for further experimental studies, because they exhibited drug-like properties, good estimated active values and formed crucial interactions. Finally, the compounds, ZINC 06843697 and ZINC00729256, could be used as novel scaffolds for further refining and optimizing.

## Supplementary Information



## Figures and Tables

**Table 1:** Analysis and comparison of pharmacophore model features.

No.	Feature name	ID	Count	Statistical frequency (%)	Structure-based pharmacophore model	Related amino acid residues
1	HBA-F 1	A1	1	7		
2	HBA-F 2	A2	1	7		
3	HBD 1	D1	11	79	✓	H21, D296, I297
4	HBD 2	D2	9	64	✓	H24, D296, I297
5	HBD 3	D3	2	14		
6	HBD 4	D4	1	7		
7	HBD 5	D5	1	7		
8	HBD 6	D6	1	7		
9	HBD 7	D7	1	7		
10	HBD 8	D8	1	7		
11	Hydrophobic 1	H1	12	86	✓	V326, M333
12	Hydrophobic 2	H2	10	71	✓	A12, L13, P257, Y260
13	Hydrophobic 3	H3	9	64	✓	W253
14	Hydrophobic 4	H4	5	36		
15	Hydrophobic 5	H5	2	14		
16	Hydrophobic 6	H6	1	7		
17	Positive ionizable 1	Pos1	9	64	✓	D296, Y325
18	Positive ionizable 1	Pos1	2	14		
19	Negative ionizable	Neg	1	7		

**Table 2:** *In vitro* antibacterial activity and cytotoxicity of final hit compounds.

Compounds	MIC (μg/mL) a					Cytotoxicity
	
	Gram-positive bacteria b		Gram-negative bacteria b			IC_50_ (μM)
	
	*S. aureus*	MRSA	*E. coli*	*P. aeruginosa*	*K. pneumoniae*	
ZINC06843697 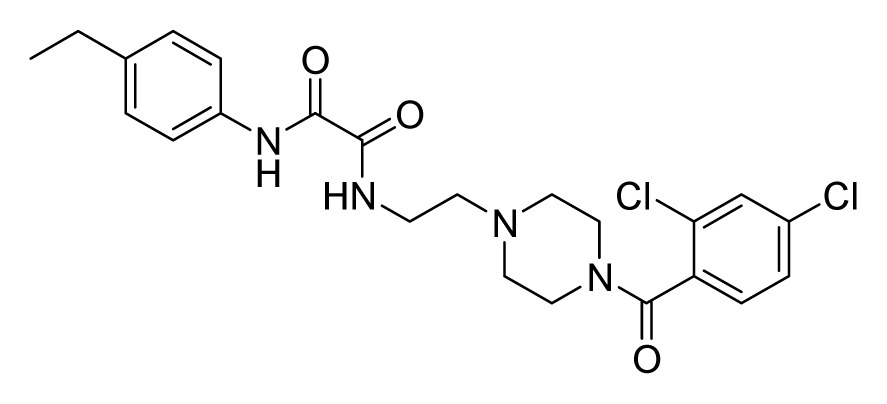	4	4	16	8	64	>20
ZINC00729256 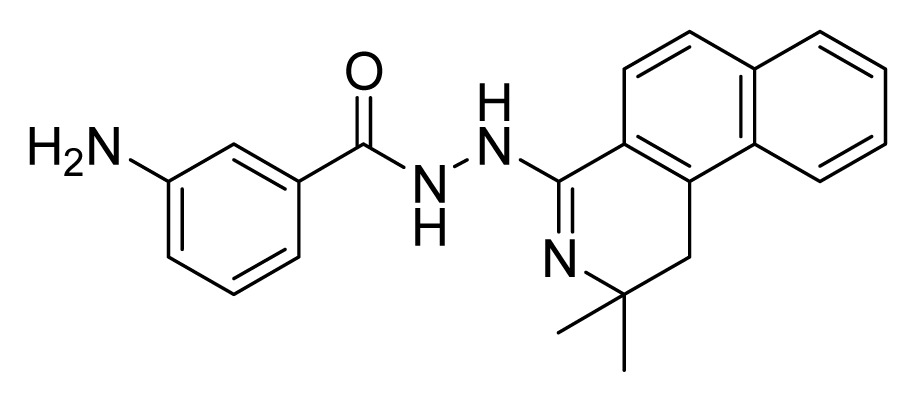	8	16	32	4	16	>20
ZINC08451958 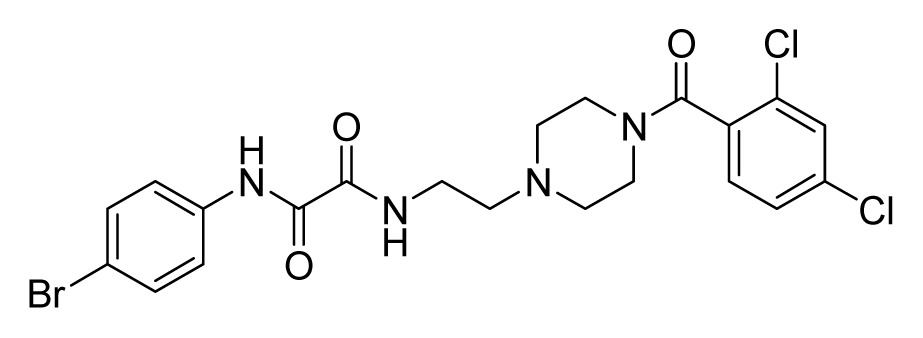	32	32	64	64	64	>20
ZINC08430843 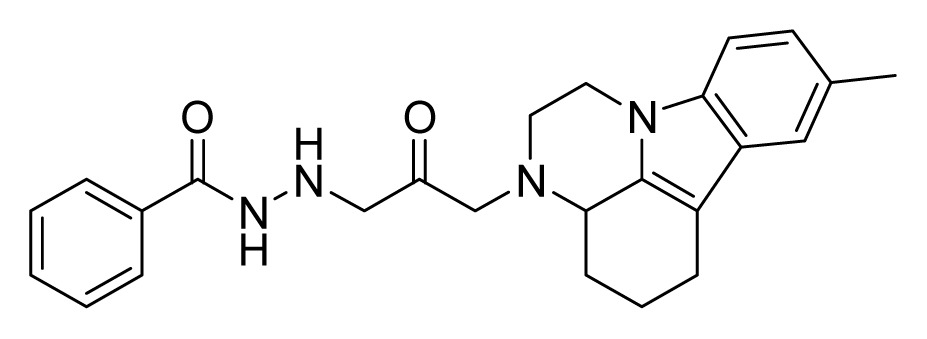	16	32	64	32	64	>20
ZINC08452043 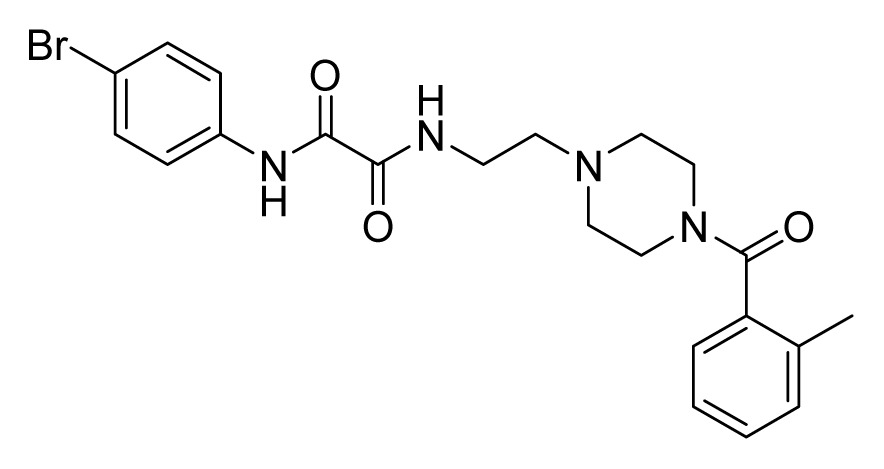	8	16	16	8	32	10.2
ZINC10312776 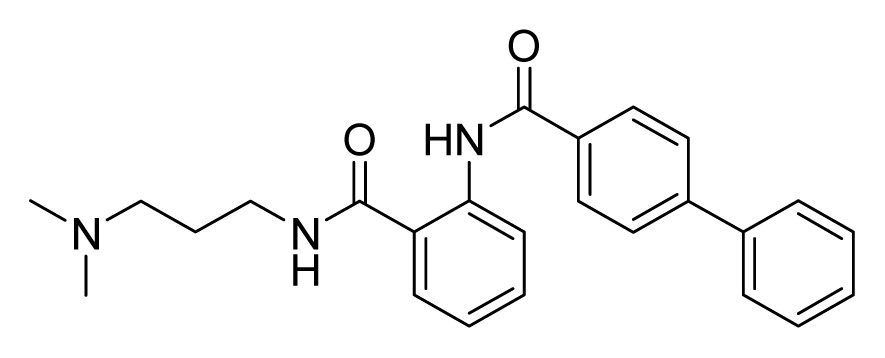	64	64	64	16	32	>20
ZINC19797060 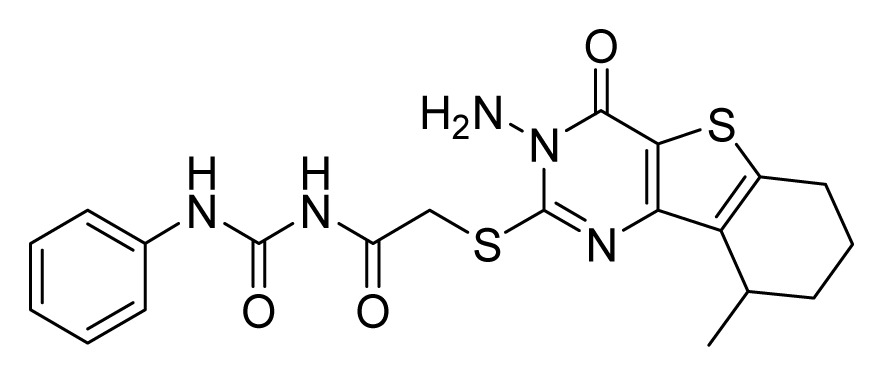	32	64	64	16	64	>20
ZINC02086896 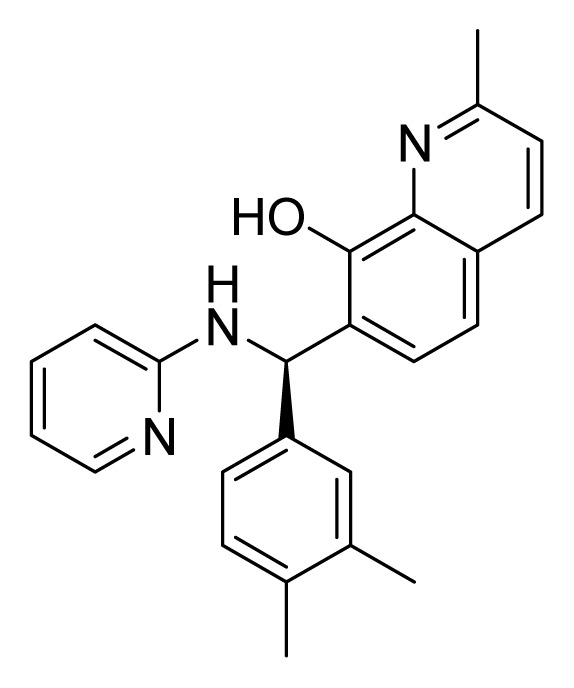	32	64	32	16	64	3.15
ZINC19922703 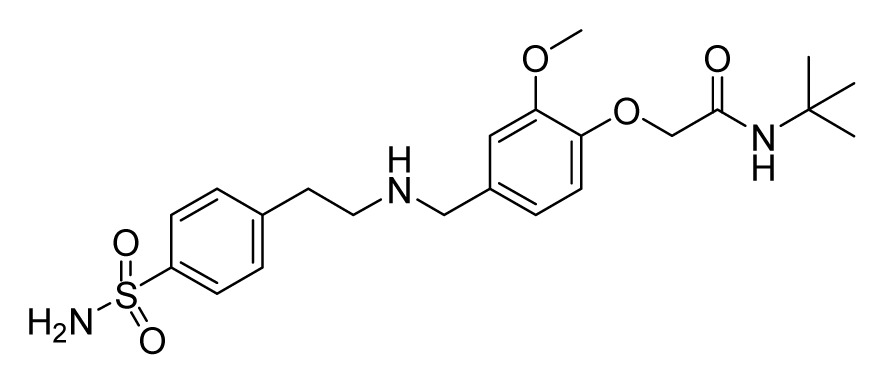	32	64	64	32	64	>20
ZINC02181060 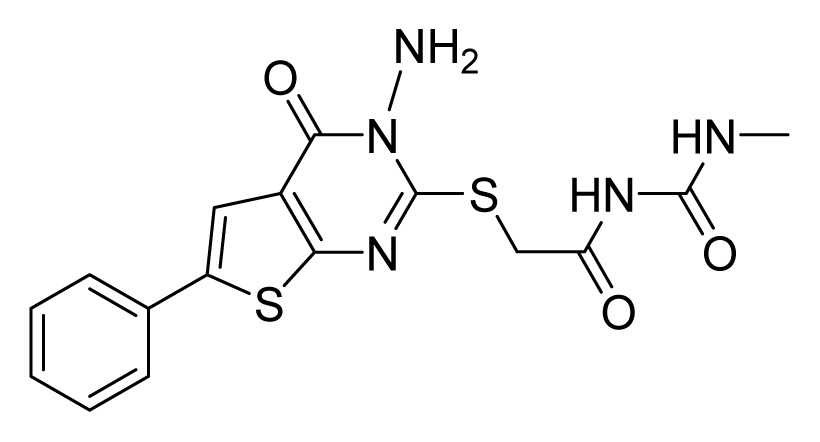	4	16	8	16	32	15.4
ZINC02424508 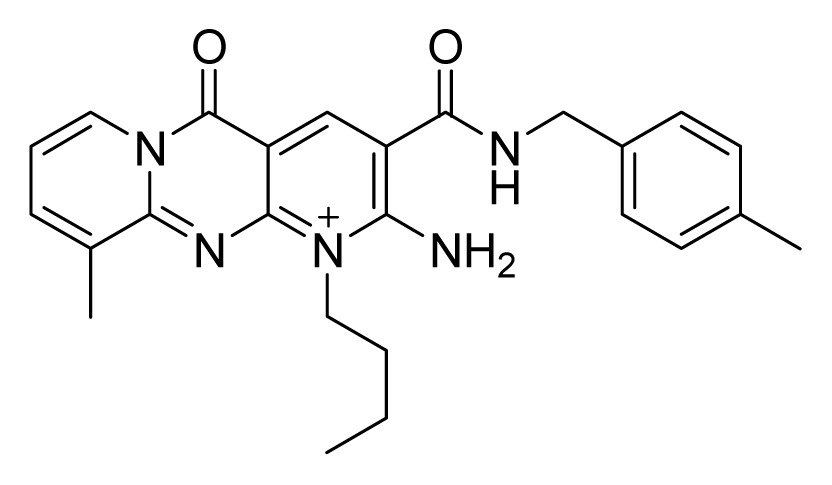	16	32	16	64	64	>20
ZINC19797059 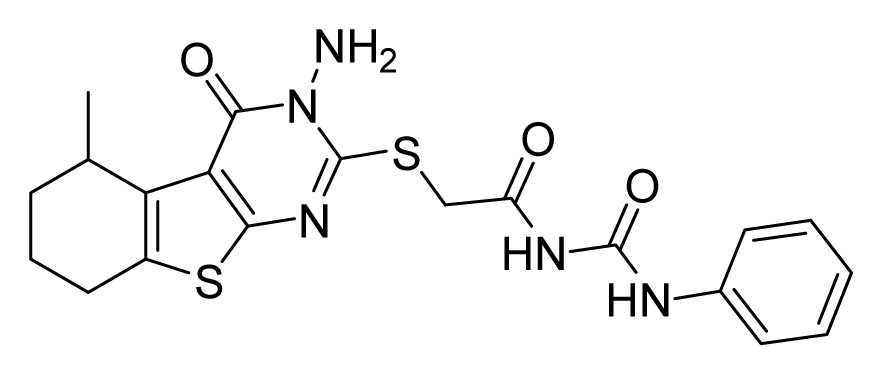	32	32	64	64	64	>20
ZINC08384332 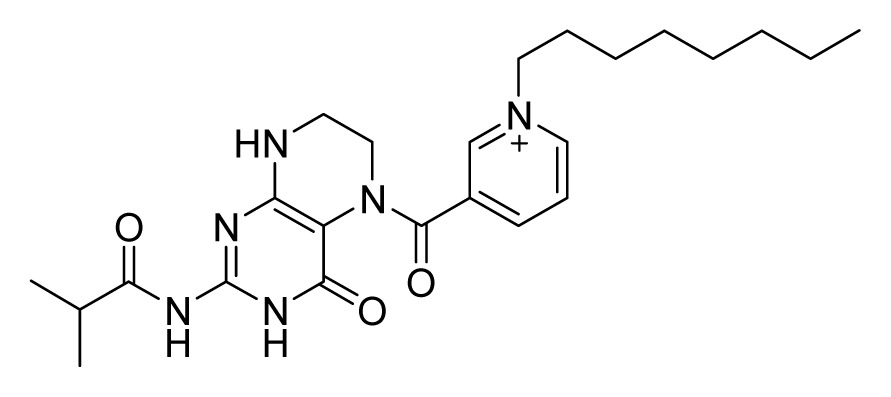	32	32	32	16	64	>20
ZINC02709613 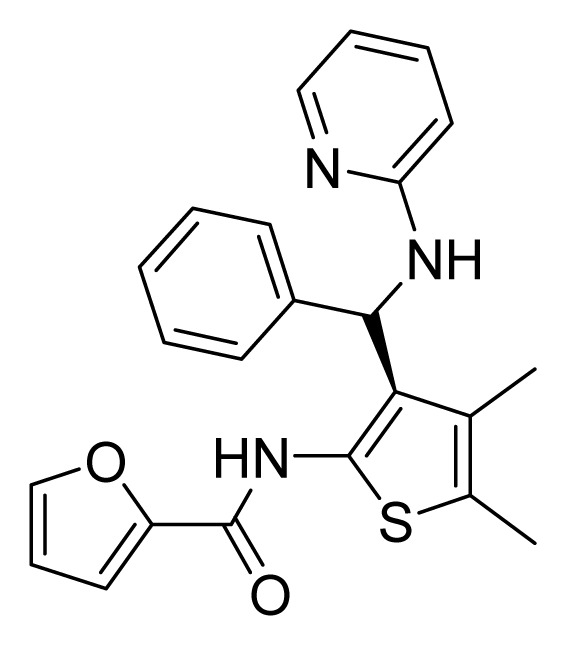	64	64	64	16	64	>20
ZINC04380079 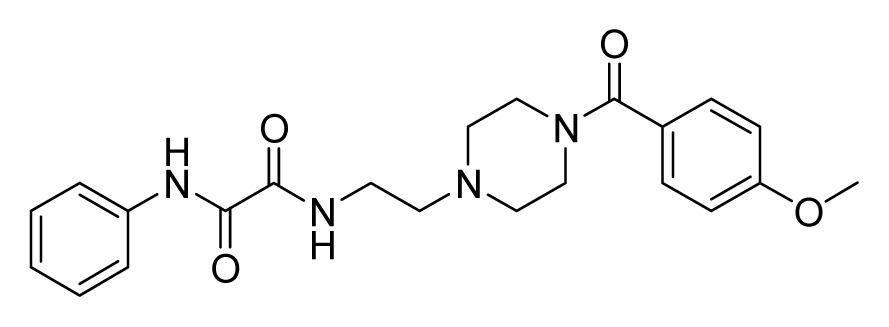	32	64	64	64	64	6.84

a Minimum inhibitory concentrations (5 × 10^5^ CFU/mL);

b definitions of organism abbreviations: *S. aureus*, *Staphylococcus aureus* ATCC 29213; MRSA, Methicillin-resistant *Staphylococcus aureus* ATCC 43300; *E. coli*, *Escherichia coli* ATCC 25922; *P. aeruginosa*, *Pseudomonas aeruginosa* ATCC 27853; *K. pneumoniae*, *Klebsiella pneumoniae* ATCC 700603.

**Table 3:** List of fourteen MetRS-inhibitor complexes used in the study.

No.	PDB	Resolution	Ligand	Release date
1	1PFY	1.93	MSP	5-27-2003
2	3KFL	2.00	ME8	10-27-2009
3	3TUN	2.55	C13	9-16-2011
4	3U1E	2.31	387	9-29-2011
5	3U1F	2.20	392	9-29-2011
6	3U1G	2.35	415	9-29-2011
7	3U1Z	2.90	43E	9-30-2011
8	3U20	2.49	44F	9-30-2011
9	4EG4	3.15	0OT	3-30-2012
10	4EG5	3.10	0OU	3-30-2012
11	4EG6	2.90	0P5	3-30-2012
12	4EG7	2.75	0P4	3-30-2012
13	4EG8	2.60	0P6	3-30-2012
14	4EGA	2.70	0P8	3-30-2012
