# Supplementary material for: Novel Hybrid Virtual Screening Protocol Based on Molecular Docking and Structure-Based Pharmacophore for Discovery of Methionyl-tRNA Synthetase Inhibitors as Antibacterial Agents
Source: Int J Mol Sci. 2013 Jul 9;14(7):14225–39. doi: 10.3390/ijms140714225 (PMC3742241; doi:10.3390/ijms140714225)
Supplement: Supplementary file 1 [file ijms-14-14225-s001.pdf]

# Supplementary Information

**Figure S1.** Superimposition of the fourteen MetRS-inhibitor complexes.

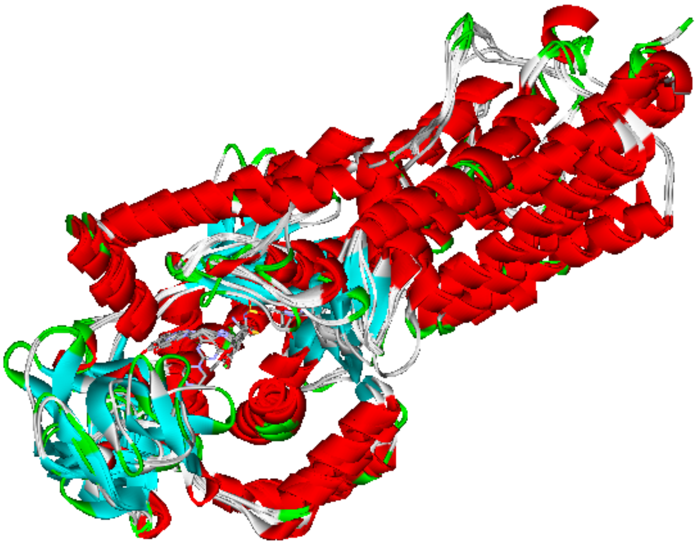

**Figure S2.** (**A**) All the chemical features of the structure-based comprehensive pharmacophore map; (**B**) View looking into the binding site of MetRS with a comprehensive map from the *N*-terminal domain. Screenshots were taken from Discovery Studio. Features of the pharmacophore models are color-coded as follows: Hydrogen bond acceptor (HBA), green; hydrogen bond donor (HBD), violet; hydrophobic (H), light blue; positive ionizable (pos), red; negative ionizable (neg), blue.

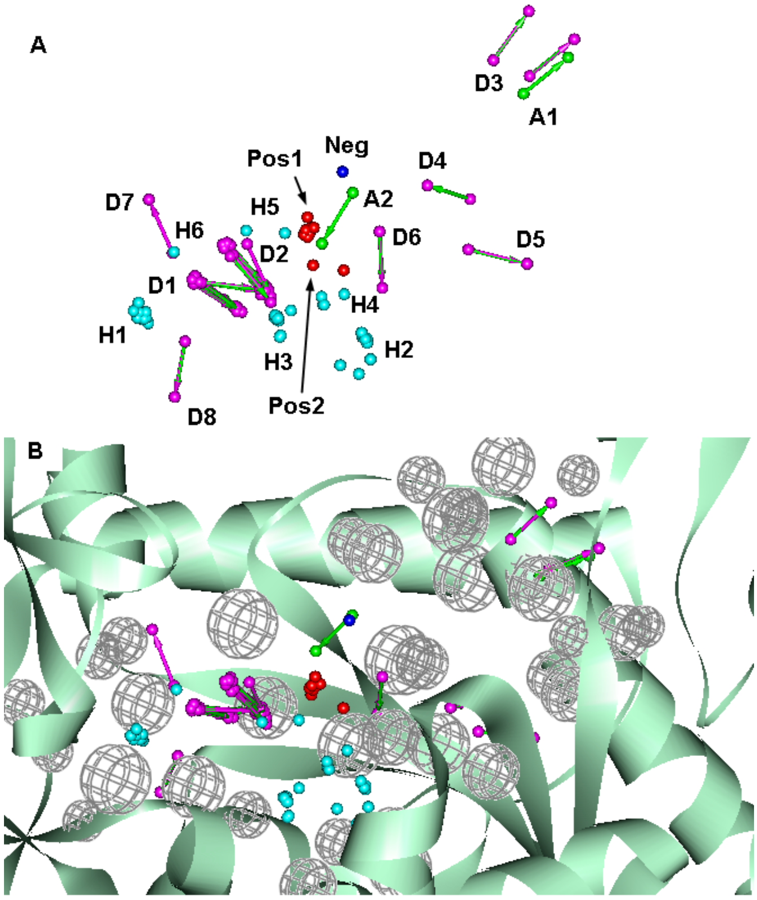

**Figure S3.** Sequence alignment result between the template protein (MetRS of *E. coli* with PDB ID: 1PFY) and the target protein, MetRS, of *S. aureus* and Human (*Homo sapiens*) represented as the subject. The blue and light blue color residues indicate the conserved residues between the template and target proteins.

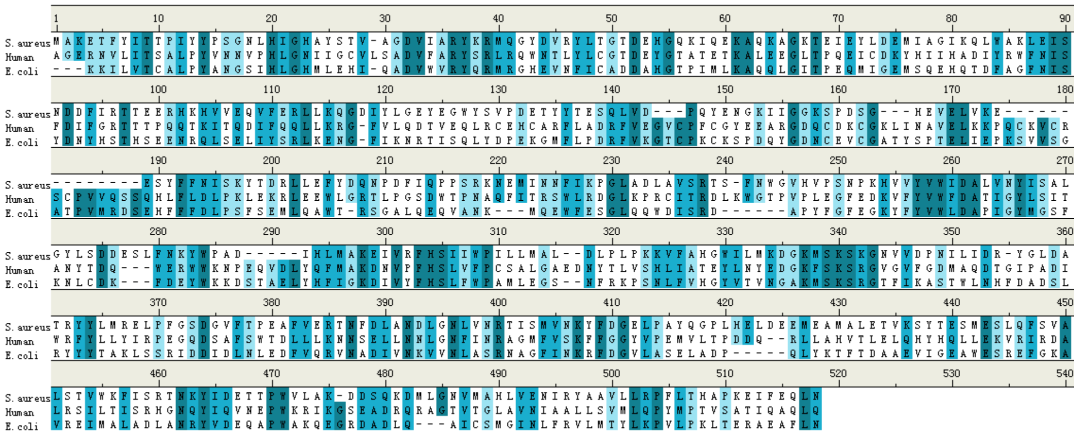


**Figure S4.** (**A**) Ramachandran plot of SaMetRS homology model; (**B**) Profile-3D verify scores per residue for the SaMetRS homology model.

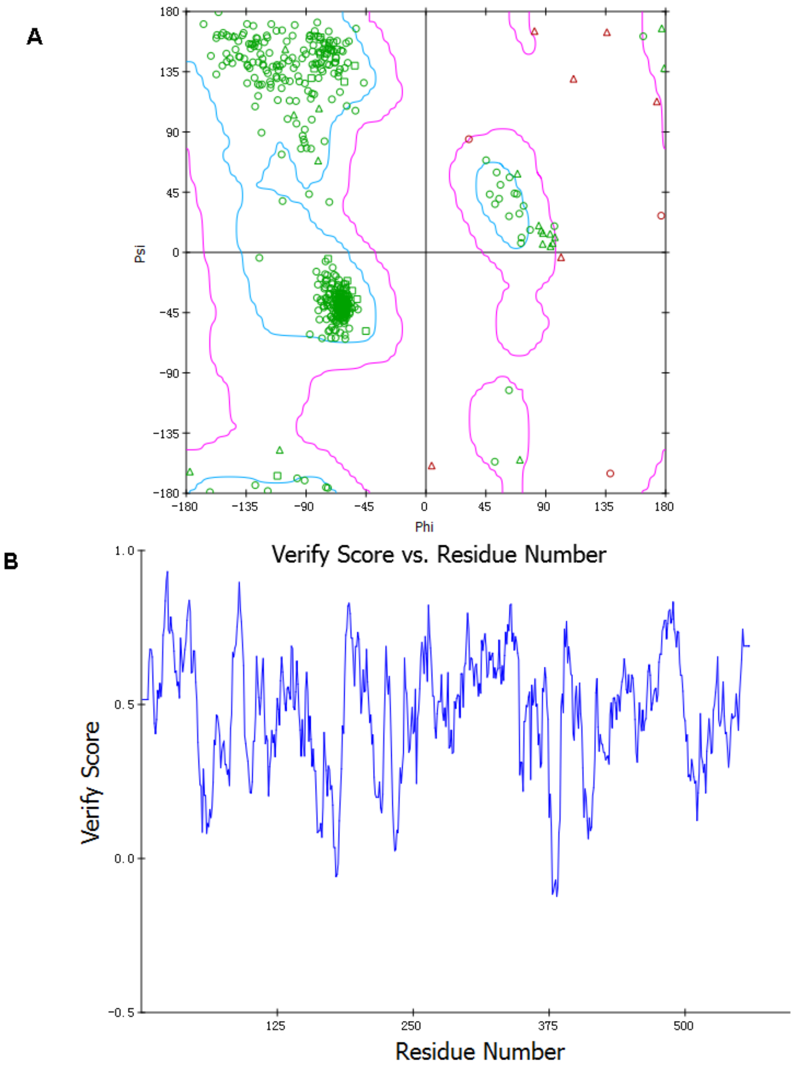

**Figure S5.** (**A**) Ramachandran plot of hMetRS homology model; (**B**) Profile-3D verify scores per residue for the hMetRS homology model.

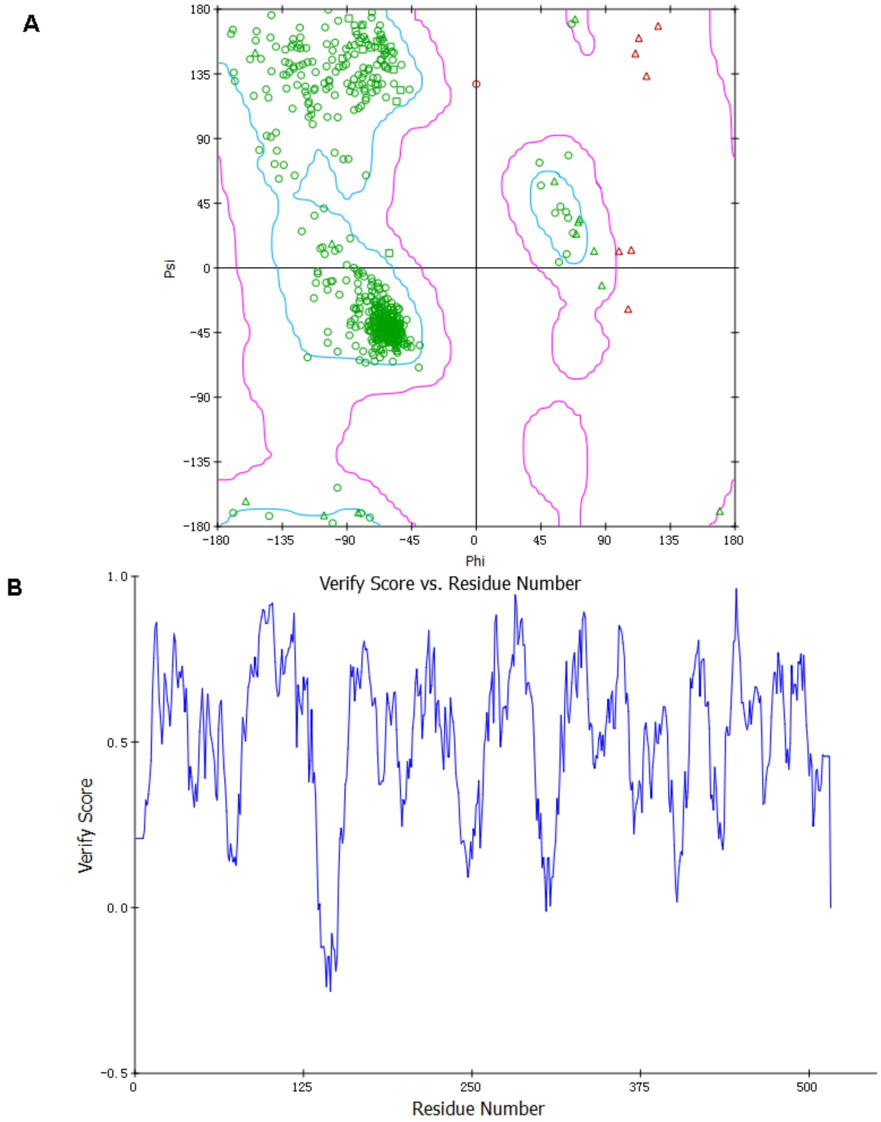


© 2013 by the authors; licensee MDPI, Basel, Switzerland. This article is an open access article distributed under the terms and conditions of the Creative Commons Attribution license (http://creativecommons.org/licenses/by/3.0/).
